# Focused ultrasound heating in brain tissue/skull phantoms with 1 MHz single-element transducer

**DOI:** 10.1007/s40477-023-00810-7

**Published:** 2023-07-30

**Authors:** Anastasia Antoniou, Nikolas Evripidou, Christakis Damianou

**Affiliations:** Department of Electrical Engineering, Computer Engineering, and Informatics, Cyprus University of Technology, 30 Archbishop Kyprianou Street, 3036 Limassol, Cyprus

**Keywords:** Focused ultrasound, Phantom, MR thermometry, Skull, Single-element, Transducer

## Abstract

**Purpose:**

The study aims to provide insights on the practicality of using single-element transducers for transcranial Focused Ultrasound (tFUS) thermal applications.

**Methods:**

FUS sonications were performed through skull phantoms embedding agar-based tissue mimicking gels using a 1 MHz single-element spherically focused transducer. The skull phantoms were 3D printed with Acrylonitrile Butadiene Styrene (ABS) and Resin thermoplastics having the exact skull bone geometry of a healthy volunteer. The temperature field distribution during and after heating was monitored in a 3 T Magnetic Resonance Imaging (MRI) scanner using MR thermometry. The effect of the skull’s thickness on intracranial heating was investigated.

**Results:**

A single FUS sonication at focal acoustic intensities close to 1580 W/cm^2^ for 60 s in free field heated up the agar phantom to ablative temperatures reaching about 90 °C (baseline of 37 °C). The ABS skull strongly blocked the ultrasonic waves, resulting in zero temperature increase within the phantom. Considerable heating was achieved through the Resin skull, but it remained at hyperthermia levels. Conversely, tFUS through a 1 mm Resin skull showed enhanced ultrasonic penetration and heating, with the focal temperature reaching 70 °C.

**Conclusions:**

The ABS skull demonstrated poorer performance in terms of tFUS compared to the Resin skull owing to its higher ultrasonic attenuation and porosity. The thin Resin phantom of 1 mm thickness provided an efficient acoustic window for delivering tFUS and heating up deep phantom areas. The results of such studies could be particularly useful for accelerating the establishment of a wider range of tFUS applications.

## Introduction

Transcranial focused ultrasound (tFUS) constitutes an evolving modality for non-invasive brain applications, including the treatment of neurodegenerative disorders such as Parkinson’s disease and essential tremor [1], the temporal disruption of the blood–brain barrier (BBB) to deliver therapeutic agents [2], as well as the stimulation of brain tissue [3]. The widespread use of tFUS has been limited for a long period of time by the challenge of accurately delivering the acoustic waves in the brain through the complex skull structure. This issue has been addressed through the development of the phased array technology, which has been a significant milestone in the process of translating tFUS applications from benchtop to bedside [4]. Another key milestone in this process was the introduction of Magnetic Resonance Imaging (MRI)-based thermometry, which allowed for precisely monitoring the FUS-induced thermal effects intra-procedurally and treating deep Central Nervous System (CNS) tissue without threatening adjacent and intervening tissues [5].

Despite the limitations of single-element transducers in terms of beam steering, they remain a valuable tool for neurotherapeutics. In recent years, low intensity tFUS has received significant attention due to its potential as a non-invasive modality for neuromodulation [6]. Successful brain stimulation by delivering low-intensity pulsed ultrasound with single-element transducers has been demonstrated in small animals [7–9], non-human primates [10], and humans [11, 12]. Single-element transducers were also proven efficient for BBB disruption (BBBD) in several animal models, including mice [13, 14] and rabbits [15], using microbubbles-enhanced pulsed FUS at frequencies of 1.5 MHz and 0.7 MHz, respectively. Lower frequencies close to 0.5 MHz were employed for successful BBBD in non-human primates [16–18] to compensate for the increased ultrasonic scattering occurring within their complex skull structure. However, the complex subject-dependent skull geometry makes it difficult to predict the amount of transmitted energy and the exact brain region affected by single-element emissions, thereby raising numerous concerns regarding clinical safety.

Image-guided numerical simulations can be used to predict ultrasonic propagation through the skull and simulate the intracranial field, thus being a valuable tool for correcting the focal point shifting and compensating for energy losses [19–22]. Such simulations are typically based on image data from computed tomography (CT) or MRI, from which the skull geometry is extracted. Yoon et al. [19] have proposed a finite-difference time domain-based simulation method employing a multi-resolution approach to model the trans-skull propagation of ultrasonic waves from single-element transducers. Performance evaluation in a sheep skull model suggests that the method can provide on-site feedback on the location, shape, and pressure profile of the focus to the user. This information is possible to allow for adjusting the transducer’s location so that the desired pressure levels are achieved at the targeted tissue with sufficient precision. A similar simulation platform was employed by Deffieux et al. [21] in an effort to examine the focalization ability of single-element transducers operating at a low frequency range of 0.3–1 MHz through both primate and human skulls in the context of FUS-mediated BBBD. In another study [20], the wave propagation by single-element emissions and the resultant intracranial energy distribution were numerically investigated in a realistic multi-tissue model of the human head to assess the feasibility of low-intensity FUS neuromodulation of the hippocampus. However, it should be noted that simulation-based guidance of tFUS may demand intensive computational resources to enable timely on-site feedback to the user.

Hydrophone-based experimental and numerical measurements were combined by Chen et al. [23], who examined the transmission of FUS from single-element transducers with frequencies of up to 1.5 MHz through human skulls. Interestingly, an exponential reduction in the transmission efficiency occurred with increasing ultrasonic frequency. An innovative virtual brain projection method has been recently proposed as another ergonomic tool for testing the behavior of tFUS beams of single-element transducers and identifying factors that may impact the effectiveness of tFUS therapy in the treatment of neurological conditions [24]. It is also worth mentioning that recently the 3D printing technology was employed in the creation of customized patient-specific holographic acoustic lenses (i.e., 3D printed plastic lenses featuring textured surfaces) to counteract the beam aberration effects induced by the varying skull thickness [25, 26]. Dedicated algorithms and simulation techniques can be used to design the digital model of the lens with the desired textured surface. This method was found to increase the energy accumulation within the targeted region by ten-fold [25], thus holding promise for tFUS thermal therapy using single-element transducers.

Recently, systems incorporating single-element transducers have been proposed for FUS-mediated BBBD under stereotactic targeting and real-time passive cavitation monitoring with the aim of enabling MRI-independent treatment sessions [27, 28]. Pouliopoulos et al. [27] presented a neuronavigation-guided system featuring a 0.25 MHz single-element transducer. Simulation studies and hydrophone-based experiments involving a human skull fragment were performed to assess the transducer’s focusing properties. As expected, the insertion of the skull fragment in the beam path resulted in considerable focal shifting and a pressure attenuation of about 45%. A similar approach was followed by Marquet et al. [28], who report successful BBBD of deep subcortical structures in monkeys with a 0.5 MHz transducer. The ultrasonic amplitude of emitted waves was increased based on pressure measurements taken in vitro to compensate for attenuation losses through the scalp and brain [28].

Tissue-mimicking phantoms have been a valuable tool in the early-stage assessment of FUS systems and emerging applications. Soft tissue is typically mimicked by a gel phantom, with agar- and PAA- based gels being widely employed for thermal studies with FUS mainly due to their ability to withstand ablative temperatures and replicate the most critical properties of biological tissues [29]. Regarding hard tissue, thermoplastic polymers have been selected for developing skull mimics by molding into dedicated patient-specific skull molds [30, 31]. The 3D printing technology has emerged as a beneficial manufacturing method with the ability to develop more complex geometries with higher precision and detail compared to molding-based manufacturing [32–34]. In this regard, accurate geometric reconstruction of the skull bone is essential for replicating the defocusing effects caused by the variable thickness and complex structure of the cranium accurately. Accordingly, in the context of examining the feasibility of delivering FUS transcranially, experiments were carried out in both simplistic and more-advanced geometrically-accurate skull models using both thermocouple and MR thermometry measurements [32, 33]. The general conclusion reached is that the skull phantoms decrease the temperatures recorded in free field substantially since the beam loses its focusing ability.

Given the recent scientific interest in transcranial FUS therapeutics using single-element transducers and the effort to establish techniques for overcoming their trans-skull steering inability, we herein present our findings on the feasibility of delivering FUS in a realistic brain tissue/skull phantom using a 1 MHz single-element spherically focused transducer. FUS sonications were performed through 3D-printed geometrically-accurate skull phantoms filled with an agar-based gel mimicking the brain tissue without any means of defocusing corrections. The temperature evolution and thermal field distribution during and after heating were monitored using MR thermometry. Skull phantoms made of two different thermoplastic materials were employed to assess the effect of ultrasonic attenuation on the thermal effects achieved within the soft tissue phantom. Furthermore, the study examined the feasibility of efficiently delivering FUS to heat up the phantom material through a 1 mm skull mimic. This technique is proposed as a potential novel approach to treat unresectable (i.e., multiple, recurrent, deep-seated, etc.) brain tumors by temporarily replacing the skull with a thin biocompatible insert to enable sufficient penetration and heating at ablative temperatures. Through these experiments, the study aims to provide insights on the practicality of using single-element transducers for tFUS in the context of thermal therapy, also given that so far, ultrasonic transmission has been mostly assessed by numerical simulations.

## Materials and methods

### Construction of brain tissue/skull phantoms

Two-compartment skull phantoms were manufactured by rapid prototyping. The skull bone model was extracted by segmentation on CT head scan images of an anonymized female volunteer. A circular piece of the temporal-parietal skull region was isolated, resulting in a two-compartment skull model. The skull model was 3D-printed using two common thermoplastic materials; Acrylonitrile Butadiene Styrene (ABS, Stratasys) and Resin (Stratasys), on the F270 and Object30 Prime 3D printers of Stratasys (Minnesota, USA), respectively. Following further processing and smoothing on the dedicated software of each printer, the phantoms were manufactured with 100% infill. The circular insert had a diameter of 60 mm and an average thickness of about 6 mm.

Another thinner skull mimic was created to account for the effect of the skull thickness on ultrasonic transmission. Specifically, the stereolithography (STL) format of the circular skull insert was processed to adjust its thickness to 1 mm through its entire surface. The thin skull mimic was 3D-printed with Resin (Stratasys) material only. The rationale behind investigating the use of a 1 mm skull insert is that by temporarily removing a small skull part and replacing it with a thin biocompatible skull insert, the FUS ablation of unresectable brain tumors by single-element emissions could be feasible. Accordingly, the benefits of single-element transducers in terms of simplicity and cost-effective over phased array transducers could be exploited through this approach.

**Fig. 1:**
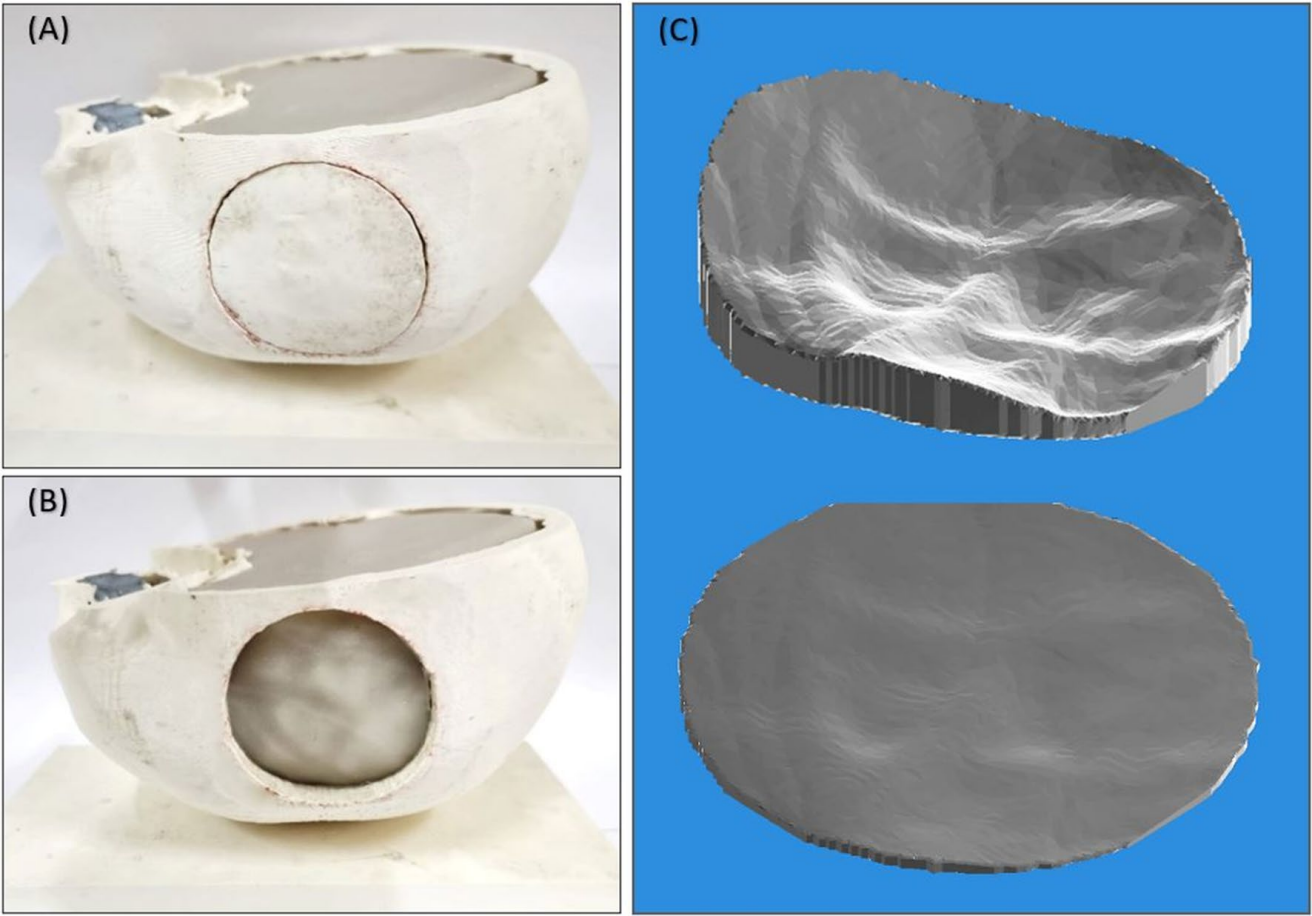
**A** The two-compartment skull phantom filled with the tissue mimicking agar gel. **B** The skull phantom with the circular insert being removed from the lateral side exposing the agar-based brain tissue phantom. **C** Comparison between the 1 mm and varying thickness Resin inserts

The brain tissue was mimicked by an agar-based gel containing a 6% weight per volume (w/v) agar (Merck KGaA, EMD Millipore Corporation, Darmstadt, Germany) and 4% w/v silicon dioxide (Sigma-Aldrich, St. Louis, Missouri, United States). The concentration of these inclusions was proven to impart the desired phantom characteristics for the specific application of thermal FUS studies, including acoustical, thermal, and MRI properties comparable to human tissues [35–37]. The ultrasonic attenuation coefficient of this phantom was previously estimated at 1.10 ± 0.09 dB/cm MHz [35]. The process for creating the gel phantom, as previously outlined by Drakos et al. [38], involved dissolving the agar and silicon dioxide powders in water. The agar solution was poured into the skull phantom and allowed to solidify, resulting in the final phantom shown in Fig. 1A. As shown in Fig. 1B, the circular skull insert can be easily removed to expose the brain-tissue phantom. Figure 1C compares the 1 mm Resin insert with that of varying thickness.

### CT imaging of the skull phantoms

Before proceeding to FUS experiments it was considered essential to investigate the existence of air pores within the phantoms, which may be introduced during 3D printing and affect the propagation of ultrasonic waves considerably. Therefore, the radiographic behavior of the ABS and Resin skull mimics was investigated. CT imaging was performed with a General Electric (GE) CT scanner (Optima 580 RT, GE Medical Systems, Wisconsin, United States) using a tube voltage of 120 kV, a tube current of 410 mA, and a slice thickness of 1.25 mm to examine if there were any voids within the Resin and ABS samples.

### FUS sonications in the phantom

FUS sonications were performed in the developed phantom with and without the circular skull insert (Fig. 1) in a 3 T MRI scanner (Magnetom Vida, Siemens Healthineers, Erlangen, Germany). The FUS transducer employed in the study was made of a spherically focused single-element piezoelectric (Piezohannas, Wuhan, China) with an operating frequency ($f$) of 1.1 MHz, a diameter ($D$) of 50 mm, a radius of curvature ($R$) of 100 mm, and an acoustic efficiency of 30%. The element was hosted in a dedicated MRI-compatible plastic housing. The transducer was supplied by an RF amplifier (AG1016, AG Series Amplifier, T & C Power Conversion, Inc., Rochester, USA) located outside of the MRI room through MR shieled cables.

**Fig. 2:**
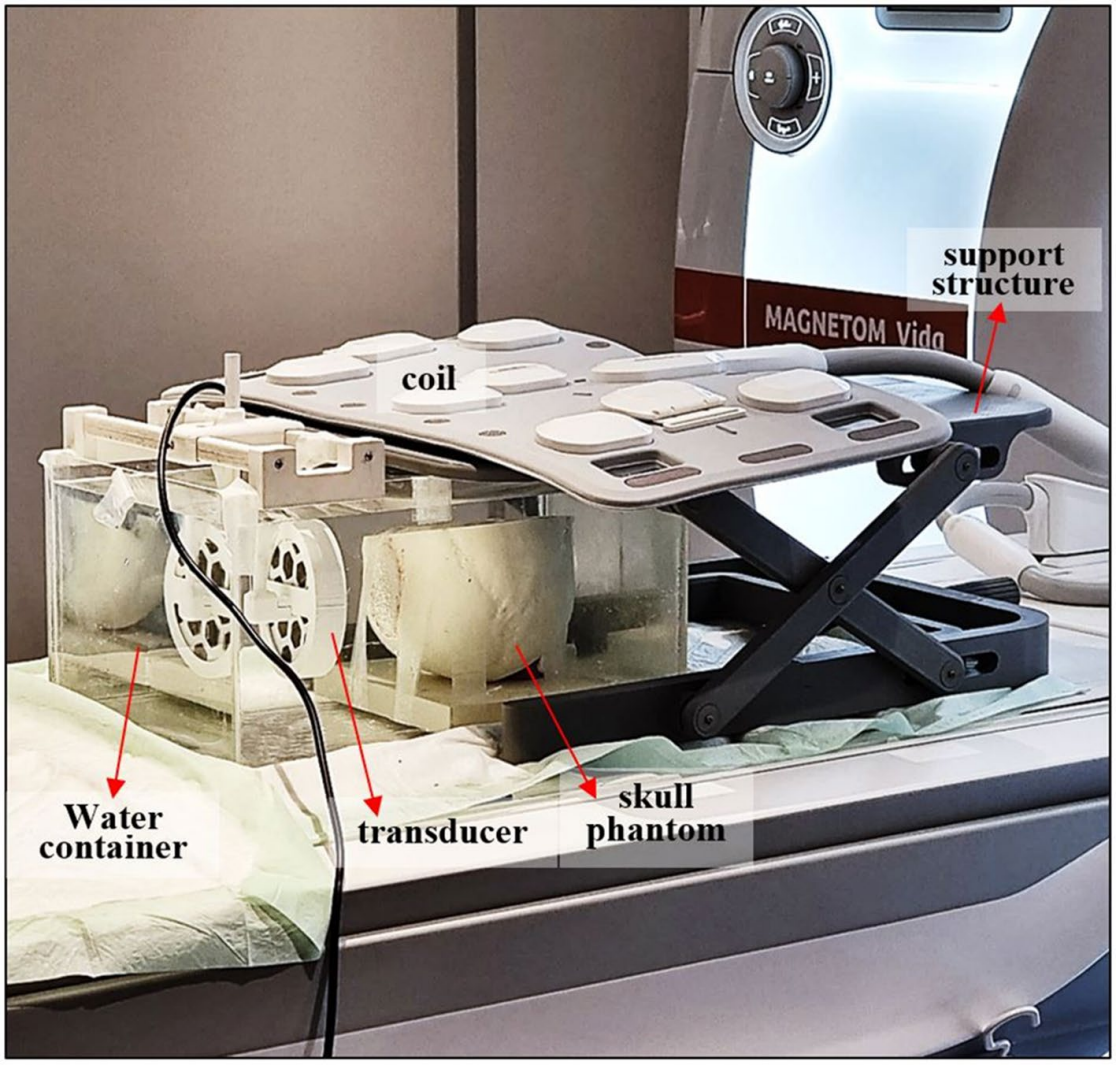
Photo of the experimental setup for FUS sonications in the brain tissue/skull phantom as arranged on the MRI table of the 3 T scanner, with the various components indicated

The experimental setup, as arranged on the MRI table, can be seen in Fig. 2. The brain tissue/skull phantom was submerged in a water tank filled with degassed and deionized water. The FUS transducer was attached to a specially designed 3D-printed holder facing toward the movable part of the phantom (circular insert). The transducer holder was attached on the top edges of the tank. The holder was able to be moved enabling adjustment of the distance between the transducer and phantom. For image acquisition, a multichannel body coil (Body18, Siemens Healthineers) was fixed on a dedicated support structure above the phantom. Caution was given not to include the transducer within the coil’s detection area to avoid interference and signal loss [39].

For all reported experiments, the distance between the transducer and phantom was adjusted so that the focal depth is 40 mm. Continuous FUS was applied at acoustic power of 90 W for 60 s. The corresponding focal intensity was calculated as the acoustic power value divided by the beam area where the ultrasound energy is concentrated (i.e., cross-sectional area at the focal point; $\uppi {\mathrm{r}}^{2}$), equaling to 1583 W/cm^2^. Notably, the focal beam diameter is typically calculated by the structural characteristics of the transducer, as $\frac{\lambda R}{D}$, where $\lambda$ is the wavelength (defined by the operating frequency and speed of sound in the medium). The temperature evolution during sonication and having a 60 s cooling time was monitored using MR thermometry. The proton resonance frequency shift (PRFS) method [5] was used for calculating the temperature changes in a Region of Interest (ROI) set within the phantom. This technique correlates the PRF change occurring during changes in the subject’s temperature with the observed differences in phase between an initial image obtained at a baseline temperature (${\varphi }_{0}$) and subsequent images obtained at various time spots ($\varphi$) during and after sonication. These phase differences ($\varphi -{\varphi }_{0}$) can be converted into temperature changes ($\Delta {\rm T}$) as follows [5]:

1 $$\Delta {\rm T} = \frac{{\varphi - \varphi_{0} }}{{\gamma \alpha {\rm B}_{0} {\rm T}{\rm E}}}$$

where $\alpha$ is the PRF change coefficient, $\gamma$ is the gyromagnetic ratio, ${\rm B}_{0}$ is the magnetic field strength (3 T), and $\mathrm{\rm T}\mathrm{\rm E}$ is the echo time. The magnitude of $\alpha$ was set at $0.0094$ ppm/°C [40, 41].

The temperature changes in the ROI were calculated based on a pixel-by-pixel analysis of the phase differences. Coronal and axial thermal maps were derived from 2D Fast Low Angle Shot (FLASH) images acquired with the following parameters: Repetition time (TR) = 25 ms, Echo time (TE) = 10 ms, Field of view (FOV) = 280 × 280 mm^2^, slice thickness = 3 mm, Number of excitations (NEX) = 1, Flip angle (FA) = 30°, Echo train length (ETL) = 1, matrix size = 96 × 96, pixel bandwidth = 250 Hz/pixel, and acquisition time/slice = 2.4 s. Color maps were produced by color-coding the measured temperatures from the minimum to the maximum value from yellow to red.

## Results

**Fig. 3:**
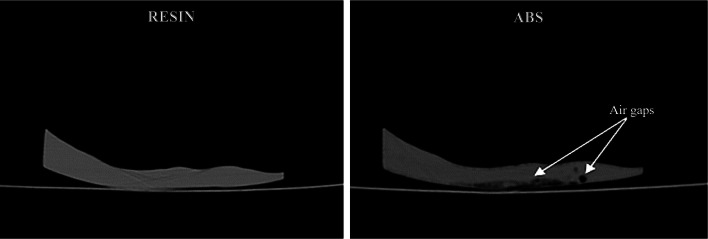
CT images of the Resin and ABS samples acquired with a tube voltage of 120 kV, current of 410 mA, and a slice thickness of 1.25 mm

Indicative CT images of the two skull mimics made of ABS and Resin are presented in Fig. 3, revealing the presence of some air-filled pores within the ABS sample. On the contrary, the Resin sample appears completely solid. This finding was useful in interpreting the results of the follow-up FUS experiments.

**Fig. 4:**
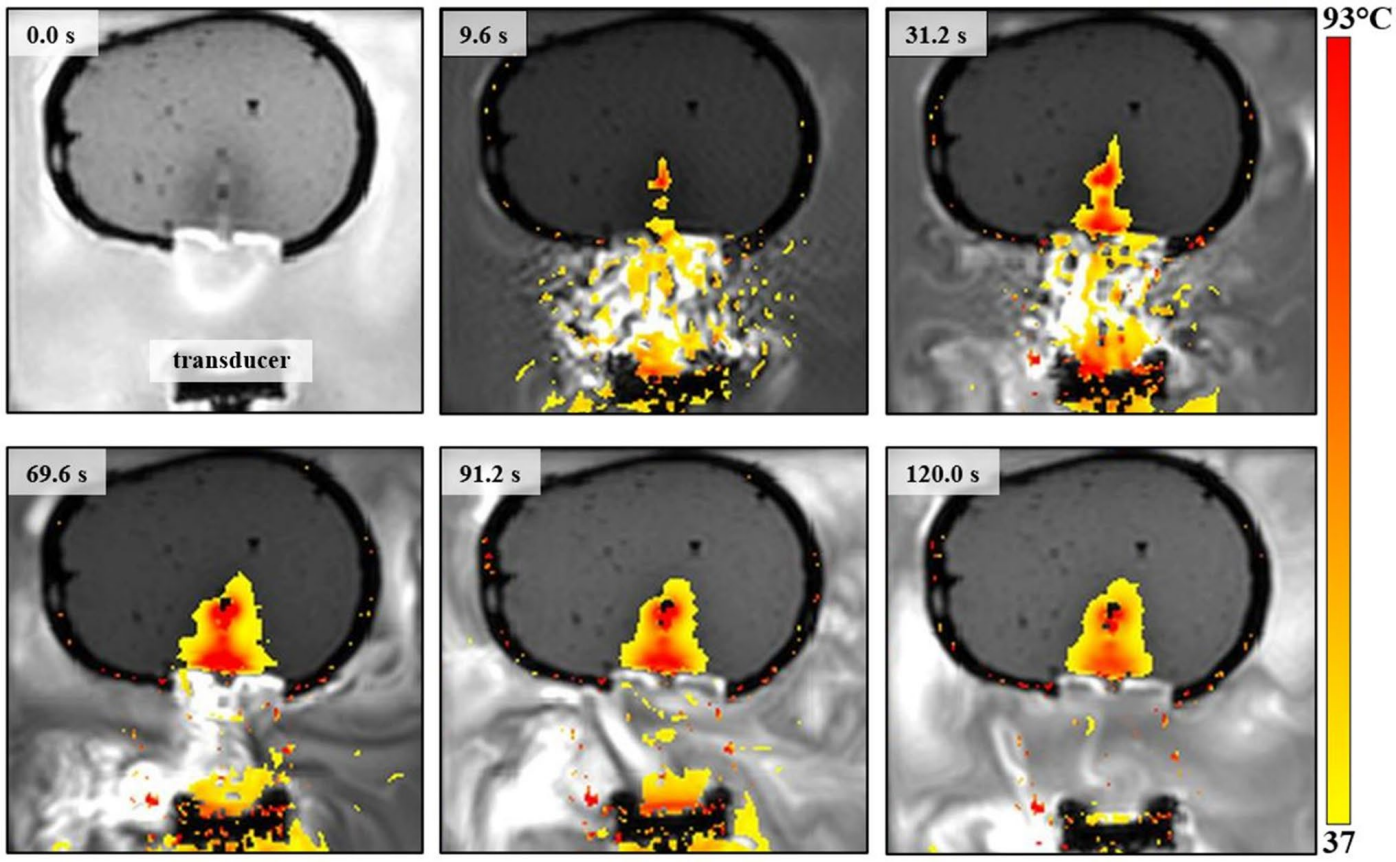
Coronal thermal maps derived from FLASH images during sonication in the phantom at acoustic power of 90 W for 60 s at a focal depth of 40 mm, without any obstacle in the beam path

**Fig. 5:**
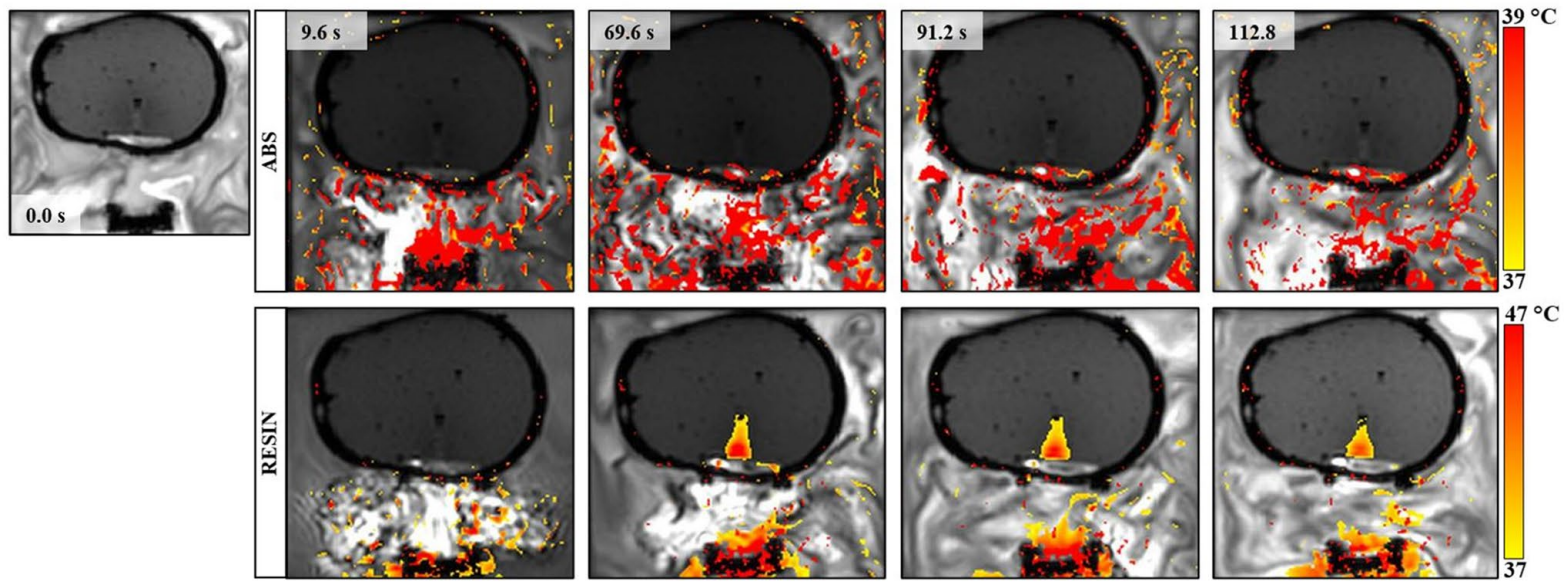
Coronal thermal maps derived from FLASH images during sonication at acoustic power of 90 W for 60 s at a focal depth of 40 mm through the ABS and Resin skull inserts

The results of FUS sonications are summarized in Table 1, along with the ultrasonic attenuation coefficients for the Resin and ABS thermoplastics, as measured using a common transmission-through immersion technique [42]. Note that a single 60 s sonication at acoustic power of 90 W, corresponding to a focal intensity of 1583 W/cm^2^, without any obstacle in the beam path (free field), as well as through the 1 mm Resin insert, heated up the agar-based material from room temperature up to ablative temperatures (> 60 °C). In fact, sonication in free field resulted in a maximum recorded focal temperature of 93 °C. Indicative thermal maps acquired at various time spots during and after heating without any obstacle intervening in the beam path are shown in Fig. 4. The corresponding results for similar sonications through the ABS and Resin skulls (of varying thickness) are shown in Fig. 5. Note that the ultrasonic waves were strongly blocked by the ABS skull resulting in zero temperature increase within the phantom volume. Conversely, detachable heating was observed in the case of the Resin skull, with the baseline temperature of 37 °C increasing to almost 47 °C at the focal area but remaining at hyperthermia levels. Note also that heating through the ABS sample resulted in a slight temperature rise of 1.8 °C in the phantom adjacent to the skull mimic surface interfering with the beam, revealing a negligible heat accumulation in the region.

**Table 1:** The focal temperature change (Δ*Τ*) recorded in the phantom using acoustical power of 90 W for 60 s at a focal depth of 40 mm with no plastic, as well as with the ABS and RESIN skull mimics intervening the beam

Skull phantom	Thickness (mm)	Δ*T* (^o^C)	Ultrasonic attenuation (dB/cm)
NO	–	55	–
ABS	6 (average)	1.8	37.7 ± 1.8
Resin	6 (average)	9.7	8.4 ± 0.2
Resin thin	1	33

**Fig. 6:**
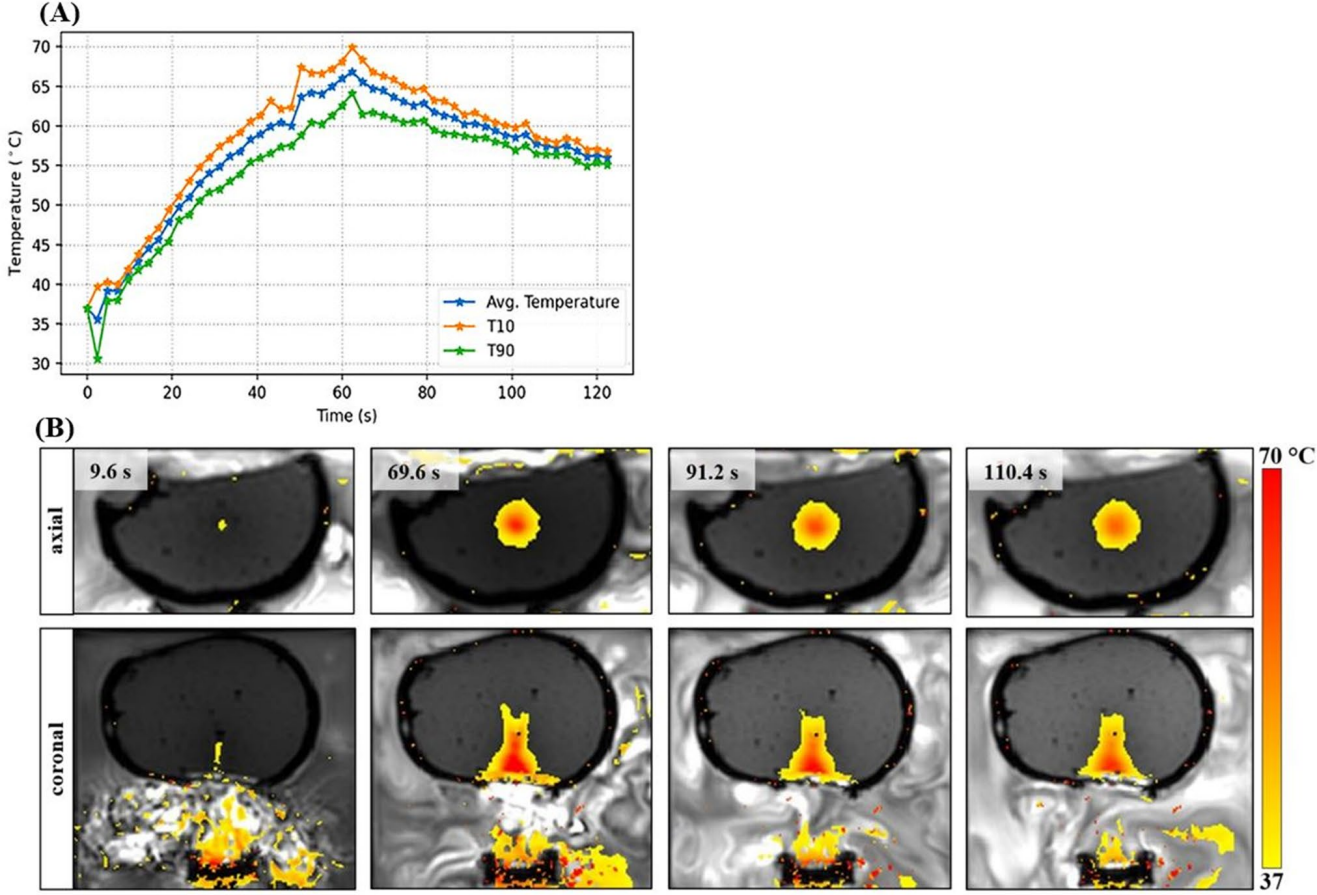
**A** Temperature increase versus time during phantom sonication throught the 1 mm Resin skull at acoustic power of 90 W for 60 s at a focal depth of 40 mm. **B** Indicative axial and coronal thermal maps acquired during sonication through the 1 mm Resin skull

The use of a thin skull phantom of 1 mm thickness provided significantly better results in terms of trans-skull ultrasonic transmission and heating of the phantom material compared to the thick one. The temperature profile of Fig. 6A reveals a maximum focal temperature of 70 °C, compared to that of 47 °C achieved by sonication through the varying thickness Resin skull. Figure 6B presents indicative thermal maps acquired in both axial and coronal planes, showing efficient beam penetration and heating of the phantom material at ablative temperatures.

## Discussion

In the current study, we examined the heating capabilities of a custom-made 1 MHz single-element spherically focused transducer through geometrically accurate skull phantoms embedding a brain-tissue mimicking material based on MR thermometry measurements. The study further provides insights on the feasibility of precisely delivering FUS through a skull mimic of 1-mm thickness as a potential method for the treatment of unresectable brain tumors. At the same time, the findings of such experiments play an essential role in the protocol optimization of MRI-compatible FUS robotic systems [43–54].

A single FUS sonication at focal acoustic intensities close to 1580 W/cm^2^ for 60 s in free field heated up the agar phantom to ablative temperatures. Ablative temperatures were also produced in the case of the 1 mm Resin insert, which allowed efficient ultrasonic penetration (Fig. 6). The focal temperature change was reduced to 60% (Δ*Τ* = 33 °C) of that achieved without any obstacle in the beam path (Δ*Τ* = 55 °C). These findings are consistent with what has been observed in prior animal research, where tFUS at frequencies close to 1 MHz was established as an efficient modality for applications in small animal models, such as mice and rabbits [14, 15], whose skull thickness is comparable to that of the thin Resin insert. In this regard, single-element transducers may also be effective for therapeutic applications in toddles through the temporal bone, which is in the order of 2 mm in thickness [55], thus potentially constituting an effective acoustic window.

On the contrary, in the presence of the varying thickness Resin insert the temperature change was decreased to about 18% (Δ*Τ* ≈ 10 °C) of that achieved in free field, whereas no heating was detected in the phantom bulk during sonication through the ABS skull. Being consistent with prior research, these findings validate that single-element transducers are incapable of effectively directing the beam through the human cranium to cause thermal heating of brain tissue unless a thorough correction method is implemented.

The Resin and ABS phantoms showed a completely different response to FUS heating. Since the defocusing effects of the varying skull thickness are considered similar for the two phantoms, this difference can be attributed to the higher ultrasonic attenuation (Table 1) and porosity of the ABS material. In fact, investigation of the radiographic behavior of the two thermoplastic materials revealed air gaps within the ABS sample. The ABS phantom was manufactured using the Fused Deposition Modeling (FDM) method, which constitutes a thermal technique that naturally incorporates pores into the manufactured specimens, thus unavoidably enhancing ultrasonic attenuation within the phantom’s interior.

There are several energy loss mechanisms affecting the ultrasonic propagation through the real skull. Intense reflections of the propagating waves occur at the interface between the skull bone and outside fluid [56, 57]. Within the skull bone, the acoustic wave is strongly scattered due to its interaction with the internal microstructure of the skull with conversions between longitudinal and shear modes taking place [56, 57]. The bone also absorbs some of the wave energy, which it then transforms into heat. Despite the complexity of quantifying the energy loss induced by each individual attenuation mechanism, it has been shown that the primary causes of attenuation are reflection, scattering, and mode conversion, whereas absorption is responsible for only a small part of the total attenuation [56]. On the contrary, in soft tissues, the wave attenuation is mostly caused by the absorption and conversion of ultrasonic energy into heat. The skull-induced spreading and defocusing of the beam reduces the penetration depth and energy deposited in tissue significantly.

The current study did not investigate the individual energy loss mechanisms occurring during propagation of ultrasonic waves through the skull phantoms. This area of investigation could be the subject of a future study. However, the study did perform a qualitative evaluation on the effect of the varying skull thickness on ultrasonic transmission and intracranial energy distribution. Although both Resin skulls allowed for sufficient beam focusing within the phantom, FUS sonication through the thin skull insert generated significantly higher temperatures (50%), heating up a larger phantom area. Furthermore, a reduction in the beam’s penetration depth was observed in the presence of the varying thickness insert, confining the heating in a narrower and shallower area of the phantom. These observations can be attributed to the acoustic aberration induced by the varying skull thickness, causing considerable energy losses and shifting of the focal spot [27].

An important consideration related to the highly aberrating nature of the human skull is the potential for thermal injuries of the skull and adjacent healthy tissues [56, 58]. The PRFS-based MR thermometry method employed in this study does not allow for measuring the skull heating directly [5]. This method relies on the detection of temperature-induced changes in the resonance frequency of water protons, and thus, a large number of protons is needed to create strong MRI signal for high quality imaging and the production of thermal maps [5]. Similarly, temperature monitoring within the thermoplastic materials that do not contain sufficient water protons is not feasible. However, the specific thermometry method can be used for monitoring the heat accumulation adjacent to the skull to assess potential damage of brain tissue [59].

In this study, there was evidence of a slight heat accumulation around the ABS skull insert. Specifically, a marginal temperature change of 1.8 °C was produced close to the skull. In the real scenario, it is expected that the complex porous structure of the cranium will more strongly attenuate the acoustic waves, potentially confining them within the skull bone, thus raising the safety concern of unwanted skull heating [60]. In this regard, an apparent limitation of the proposed skull model is its solid infill, which makes it a very simplistic model in comparison to the real cranium consisting of both cortical and cancellous bone compartments. Notably, studies have showed that during trans-skull heating, active cooling of the skull surface is essential to protecting the bone and surrounding tissues from thermal damages [56]. The Insightec’s Exablate Neuro; currently the only FDA-approved MRI-guided FUS device for brain applications, performs active cooling of the cranium and scalp by water circulation [61]. In addition, the transmission efficacy can be enhanced by selecting a proper transducer frequency, further contributing to the mitigation of such risks [23].

Phased array ultrasonic transducers are predominantly used in the context of clinical tFUS since they allow for targeting deep brain regions with the required precision to produce the desired therapeutic effects without harming healthy tissue, thus meeting the clinical requirements [62]. They also contribute towards delivering the ultrasonic energy over a large skull area, thus reducing the possibility for excessive heat accumulation in the skull [63]. However, it could be argued that their main limitation compared to single-element transducers is their increased complexity and expensiveness, as well as the need to use advanced signal processing algorithms to control the individual elements of the array [62].

The present findings provide initial evidence on the feasibility of the proposed approach of treating recurrent, multiple, or deep-seated brain tumors that cannot be removed surgically by FUS ablation through a 1 mm biocompatible skull insert. Temporal replacement of a small skull part with a 1 mm skull mimic is expected to allow the development of high temperatures of up to 90 °C within the tumor and repeated therapies to be performed. This approach exploits the unique advantages of single-element transducers (less expensive, more ergonomic, etc.) over phased arrays, thus addressing the concerns regarding insufficient trans-skull ultrasonic penetration and focal temperature increase. These benefits come at the cost of performing a small craniotomy, which is still far less invasive compared to the standard surgical therapy. Remarkably, the highest temperatures achieved through intact skull with phased arrays have been so far limited to around 60 °C [64]. A more comprehensive preclinical experimentation is required to demonstrate reproducibility of these promising results and the clinical potential of the proposed approach.

In conclusion, a variety of tFUS applications has been successfully performed using single-element FUS transducers mostly in the preclinical setting. The wider adoption and clinical translation of this modality is limited by challenges related to inefficient trans-skull ultrasonic transmission and relevant safety concerns. Although further research is needed to fully exploit the potential of this modality, the preclinical investigation of transcranial ultrasonic propagation from single-element transducers was limited to numerical studies in the context of low intensity tFUS neuromodulation. Therefore, experimental studies involving anthropomorphic phantoms such as the current one could be a valuable tool for accelerating the establishment of a wider range of tFUS applications (including tFUS ablation) potentially working supplementary to numerical studies.

## Data Availability

All data generated or analysed in the present study are available from the corresponding author on reasonable request.

## References

[1] Nicodemus NE, Becerra S, Kuhn TP, Packham HR, Duncan J, Mahdavi K (2019). Focused transcranial ultrasound for treatment of neurodegenerative dementia. Alzheimer’s Dement Transl Res Clin Interv.

[2] Wasielewska JM, White AR (2022). Focused ultrasound-mediated drug delivery in humans—a path towards translation in neurodegenerative diseases. Pharm Res.

[3] Zhang T, Pan N, Wang Y, Liu C, Hu S (2021). Transcranial focused ultrasound neuromodulation: a review of the excitatory and inhibitory effects on brain activity in human and animals. Front Hum Neurosci.

[4] Hynynen K, Clement GT, McDannold N, Vykhodtseva N, King R, White PJ (2004). 500-Element ultrasound phased array system for noninvasive focal surgery of the brain: a preliminary rabbit study with ex vivo human skulls. Magn Reson Med.

[5] Rieke V, Pauly KB (2008). MR thermometry. J Magn Reson Imaging.

[6] Arulpragasam AR, der WoutFrank M, Barredo J, Faucher CR, Greenberg BD, Philip NS 2022. Low Intensity Focused Ultrasound for Non-invasive and Reversible Deep Brain Neuromodulation—A Paradigm Shift in Psychiatric Research. Front Psychiatry. Doi: 10.3389/fpsyt.2022.825802. 10.3389/fpsyt.2022.825802 PMC8907584 35280168

[7] Kima H, Chiu A, Lee SD, Fischer K, Yoo S-S (2014). Focused ultrasound-mediated non-invasive brain stimulation: examination of sonication parameters. Brain Stimul.

[8] Yoo S-S, Bystritsky A, Lee J-H, Zhang Y, Fischer K, Min B-K, McDannold NJ, Pascual-Leone A, Jolesz FA (2011). Focused ultrasound modulates region-specific brain activity. Neuroimage.

[9] Kim H, Park MY, Lee SD, Lee W, Chiua A, Yoo S-S (2015). Suppression of EEG visual-evoked potentials in rats via neuromodulatory focused ultrasound. NeuroReport.

[10] Wattiez N, Constans C, Deffieux T, Daye PM, Tanter M, Aubry J-F (2017). Transcranial ultrasonic stimulation modulates single-neuron discharge in macaques performing an antisaccade task. Brain Stimul.

[11] Lee W, Kim HC, Jung Y, Chung YA, Song IU, Lee JH (2016). Transcranial focused ultrasound stimulation of human primary visual cortex. Sci Rep.

[12] Lee W, Kim S, Kim B, Lee C, Chung YA, Kim L (2017). Non-invasive transmission of sensorimotor information in humans using an EEG/focused ultrasound brain-to-brain interface. PLoS One.

[13] Choi JJ, Selert K, Gao Z, Samiotaki G, Baseri B, Konofagou EE (2011). Noninvasive and localized blood—brain barrier disruption using focused ultrasound can be achieved at short pulse lengths and low pulse repetition frequencies. J Cereb Blood Flow Metab.

[14] Wang S, Samiotaki G, Olumolade O, Feshitan JA, Konofagou EE (2014). Microbubble type and distribution dependence of focused ultrasound-induced blood-brain barrier opening. Ultrasound Med Biol.

[15] Hynynen K, Mcdannold N, Sheikov NA, Jolesz FA, Vykhodtseva N (2005). Local and reversible blood–brain barrier disruption by noninvasive focused ultrasound at frequencies suitable for trans-skull sonications. Neuroimage.

[16] Samiotaki G, Karakatsani ME, Buch A, Papadopoulos S, Wu SY, Jambawalikar S (2017). Pharmacokinetic analysis and drug delivery efficiency of the focused ultrasound-induced blood-brain barrier opening in non-human primates. Magn Reson Imaging.

[17] Wu SY, Aurup C, Sanchez CS, Grondin J, Zheng W, Kamimura H (2018). Efficient blood-brain barrier opening in primates with neuronavigation-guided ultrasound and real-time acoustic mapping. Sci Rep.

[18] Karakatsani ME, Samiotaki G, Downs ME, Ferrera VP, Konofagou EE (2017). Targeting effects on the volume of the focused ultrasound induced blood-brain barrier opening in non-human primates in vivo. IEEE Trans Ultrason Ferroelectr Freq Control.

[19] Yoon K, Lee W, Croce P, Cammalleri A, Yoo S-S (2019). Multi-resolution simulation of focused ultrasound propagation through ovine skull from a single-element transducer. Phys Med Biol.

[20] Huang Y, Wen P, Song B, Li Y (2022). Numerical investigation of the energy distribution of low-intensity transcranial focused ultrasound neuromodulation for hippocampus. Ultrasonics.

[21] Deffieux T, Konofagou EE (2010). Numerical study of a simple transcranial focused ultrasound system applied to blood-brain barrier opening. IEEE Trans Ultrason Ferroelectr Freq Control.

[22] Seo H, Huh H, Lee EH, Park J (2022). Numerical evaluation of the effects of transducer displacement on transcranial focused ultrasound in the rat brain. Brain Sci.

[23] Chen M, Peng C, Wu H, Huang CC, Kim T, Traylor Z (2023). Numerical and experimental evaluation of low-intensity transcranial focused ultrasound wave propagation using human skulls for brain neuromodulation. Med Phys.

[24] Brinker ST, Preiswerk F, McDannold NJ, Parker KL, Mariano TY (2019). Virtual brain projection for evaluating trans-skull beam behavior of transcranial ultrasound devices. Ultrasound Med Biol.

[25] Maimbourg G, Houdouin A, Deffieux T, Tanter M (2018). 3D-printed adaptive acoustic lens as a disruptive technology for transcranial ultrasound therapy using single-element transducers. Phys Med Biol.

[26] Ferri M, Bravo JM, Redondo J, Sanchez-Perez JV (2019). Enhanced numerical method for the design of 3-D-printed holographic acoustic lenses for aberration correction of single-element transcranial focused ultrasound. Ultrasound Med Biol.

[27] Pouliopoulos AN, Wu SY, Burgess MT, Karakatsani ME, Kamimura HAS, Konofagou EE (2020). A clinical system for non-invasive blood-brain barrier opening using a neuronavigation-guided single-element focused ultrasound transducer. Ultrasound Med Biol.

[28] Marquet F, Teichert T, Wu SY, Tung YS, Downs M, Wang S (2014). Real-time, transcranial monitoring of safe blood-brain barrier opening in non-human primates. PLoS One.

[29] Antoniou A, Damianou C (2022). MR relaxation properties of tissue-mimicking phantoms. Ultrasonics.

[30] Mackle EC, Shapey J, Maneas E, Saeed SR, Bradford R, Ourselin S (2020). Patient-specific polyvinyl alcohol phantom fabrication with ultrasound and x-ray contrast for brain tumor surgery planning. J Vis Exp.

[31] Tan ETW, Ling JM, Dinesh SK (2016). The feasibility of producing patient-specific acrylic cranioplasty implants with a low-cost 3D printer. J Neurosurg.

[32] Hadjisavvas V, Mylonas N, Ioannides K, Damianou C (2012). An MR-compatible phantom for evaluating the propagation of high intensity focused ultrasound through the skull. AIP Conf Proc.

[33] Menikou G, Dadakova T, Pavlina M, Bock M, Damianou C (2015). MRI compatible head phantom for ultrasound surgery. Ultrasonics.

[34] Menikou G, Yiannakou M, Yiallouras C, Ioannides C, Damianou C (2018). MRI-compatible breast/rib phantom for evaluating ultrasonic thermal exposures. Int J Med Robot Comput Assist Surg.

[35] Drakos T, Antoniou A, Evripidou N, Alecou T, Giannakou M, Menikou G (2021). Ultrasonic attenuation of an agar, silicon dioxide, and evaporated milk gel phantom. J Med Ultrasound.

[36] Menikou G, Damianou C (2017). Acoustic and thermal characterization of agar based phantoms used for evaluating focused ultrasound exposures. J Ther Ultrasound.

[37] Antoniou A, Georgiou L, Christodoulou T, Panayiotou N, Ioannides C, Zamboglou N (2022). MR relaxation times of agar-based tissue-mimicking phantoms. J Appl Clin Med Phys.

[38] Drakos T, Giannakou M, Menikou G, Constantinides G, Damianou C (2021). Characterization of a soft tissue-mimicking agar/wood powder material for MRgFUS applications. Ultrasonics.

[39] Antoniou A, Georgiou L, Evripidou N, Ioannides C, Damianou C (2022). Challenges regarding MR compatibility of an MRgFUS robotic system. J Magn Reson.

[40] Peters RD, Hinks RS, Henkelman RM (1999). Heat-source orientation and geometry dependence in proton-resonance frequency shift magnetic resonance thermometry. Magn Reson Med.

[41] Bing C, Staruch R, Tillander M, Köhler MO, Mougenot C, Ylihautala M (2017). Drift correction for accurate PRF shift MR thermometry during mild hyperthermia treatments with MR-HIFU. Int J Hyperth.

[42] Antoniou A, Damianou C (2023). Feasibility of ultrasonic heating through skull phantom using single-element transducer. J Med Ultrasound.

[43] Epaminonda E, Drakos T, Kalogirou C, Theodoulou M, Yiallouras C, Damianou C (2016). MRI guided focused ultrasound robotic system for the treatment of gynaecological tumors. Int J Med Robot Comput Assist Surg.

[44] Yiannakou M, Menikou G, Yiallouras C, Ioannides C, Damianou C (2017). MRI guided focused ultrasound robotic system for animal experiments. Int J Med Robot Comput Assist Surg.

[45] Menikou G, Yiallouras C, Yiannakou M, Damianou C (2017). MRI-guided focused ultrasound robotic system for the treatment of bone cancer. Int J Med Robot Comput Assist Surg.

[46] Antoniou A, Giannakou M, Evripidou N, Evripidou G, Spanoudes K, Menikou G (2021). Robotic system for magnetic resonance guided focused ultrasound ablation of abdominal cancer. Int J Med Robot Comput Assist Surg.

[47] Drakos T, Giannakou M, Menikou G, Filippou A, Evripidou N, Spanoudes K (2021). MRI-guided focused ultrasound robotic system for preclinical use. J Vet Med Anim Sci.

[48] Damianou C, Giannakou M, Menikou G, Ioannou L (2020). Magnetic resonance imaging-guided focused ultrasound robotic system with the subject placed in the prone position. Digit Med.

[49] Antoniou A, Giannakou M, Evripidou N, Stratis S, Pichardo S, Damianou C (2022). Robotic system for top to bottom MRgFUS therapy of multiple cancer types. Int J Med Robot Comput Assist Surg.

[50] Giannakou M, Antoniou A, Damianou C (2023). Preclinical robotic device for magnetic resonance imaging guided focussed ultrasound. Int J Med Robot Comput Assist Surg.

[51] Antoniou A, Giannakou M, Georgiou E, Kleopa KA, Damianou C (2022). Robotic device for transcranial focussed ultrasound applications in small animal models. Int J Med Robot Comput Assist Surg.

[52] Filippou A, Evripidou N, Damianou C (2023). Robotic system for magnetic resonance imaging-guided focused ultrasound treatment of thyroid nodules. Int J Med Robot.

[53] Giannakou M, Drakos T, Menikou G, Evripidou N, Filippou A, Spanoudes K (2021). Magnetic resonance image-guided focused ultrasound robotic system for transrectal prostate cancer therapy. Int J Med Robot.

[54] Yiallouras C, Yiannakou M, Menikou G, Damianou C (2017). A multipurpose positioning device for magnetic resonance imaging-guided focused ultrasound surgery. Digit Med.

[55] Rahne T, Svensson S, Lagerkvist H, Holmberg M, Plontke SK, Wenzel C (2021). Assessment of temporal bone thickness for implantation of a new active bone-conduction transducer. Otol Neurotol.

[56] Pinton G, Aubry J, Bossy E, Muller M, Pernot M (2012). Attenuation, scattering, and absorption of ultrasound in the skull bone. Med Phys.

[57] Fry FJ, Barger JE (1978). Acoustical properties of the human skull. J Acoust Soc Am.

[58] Pichardo S, Sin VW, Hynynen K (2011). Multi-frequency characterization of the speed of sound and attenuation coefficient for longitudinal transmission of freshly excised human skulls. Phys Med Biol.

[59] McDannold N, King RL, Hynynen K (2004). MRI monitoring of heating produced by ultrasound absorption in the skull: in vivo study in pigs. Magn Reson Med.

[60] Chen M, Peng C, Kim T, Chhatbar P, Muller M, Feng W, et al. (2021) Biosafety of low-intensity pulsed transcranial focused ultrasound brain stimulation: a human skull study. Health Monitoring of Structural and Biological Systems XV, p. 34. 10.1117/12.2582487

[61] Sheehan J, Monteith S, Wintermark M (2015). Transcranial MR-guided focused ultrasound: a review of the technology and neuro applications. AJR Am J Roentgenol.

[62] Hynynen K, Jones RM (2016). Image-guided ultrasound phased arrays are a disruptive technology for non-invasive therapy. Phys Med Biol.

[63] Sun J, Hynynen K (1999). The potential of transskull ultrasound therapy and surgery using the maximum available skull surface area. J Acoust Soc Am.

[64] Wu P, Lin W, Li KH, Lai HC, Lee MT, Tsai KWK (2021). Focused ultrasound thalamotomy for the treatment of essential tremor: A 2-Year outcome study of Chinese people. Front Aging Neurosci.

